# The Application of Spray-Dried and Reconstituted Flaxseed Oil Cake Extract as Encapsulating Material and Carrier for Probiotic *Lacticaseibacillus rhamnosus* GG

**DOI:** 10.3390/ma14185324

**Published:** 2021-09-15

**Authors:** Łukasz Łopusiewicz, Elżbieta Bogusławska-Wąs, Emilia Drozłowska, Paulina Trocer, Alicja Dłubała, Kinga Mazurkiewicz-Zapałowicz, Artur Bartkowiak

**Affiliations:** 1 Center of Bioimmobilisation and Innovative Packaging Materials, Faculty of Food Sciences and Fisheries, West Pomeranian University of Technology Szczecin, Janickiego 35, 71-270 Szczecin, Poland; emilia_drozlowska@zut.edu.pl (E.D.); paulina.trocer@zut.edu.pl (P.T.); Artur-Bartkowiak@zut.edu.pl (A.B.); 2 Department of Applied Microbiology and Human Nutrition Physiology, West Pomeranian University of Technology Szczecin, Papieża Pawła VI 3, 71-899 Szczecin, Poland; elzbieta.boguslawska-was@zut.edu.pl (E.B.-W.); alicja.dlubala@zut.edu.pl (A.D.); 3 Department of Hydrobiology, Ichthyology and Biotechnology of Reproduction, West Pomeranian University of Technology in Szczecin, Kazimierza Królewicza 4, 71-899 Szczecin, Poland; kinga.mazurkiewicz-zapalowicz@zut.edu.pl

**Keywords:** proteins, polysaccharides, plant extracts, probiotics, *Lacticaseibacillus rhamnosus* GG, spray drying, flaxseed oil cake, simulated gastrointestinal tract

## Abstract

Agro-industrial by-products are promising source of biopolymers, including proteins and polysaccharides. This study was designed to evaluate the flaxseed oil cake extract (FOCE) as natural encapsulating material and carrier for probiotic *Lacticaseibacillus rhamnous* GG (LGG). The powders were obtained using three spray drying inlet temperatures (110 °C, 140 °C, 170 °C), and reconstituted. The influence of temperature on water activity, morphology, chemical composition, flowability and cohesiveness of the powders was estimated. For all variants, the survival of bacteria during spray drying, and simulated passage through the gastrointestinal tract was evaluated. The preservation of LGG probiotic features such as cholesterol reduction, hydrophobicity and adhesion to mucin were examined. Results revealed that all physicochemical and functional characteristics of the powders were affected by the inlet temperature. This study demonstrated that FOCE is an appropriate matrix for spray drying (due to flaxseed proteins and polysaccharides) providing high survivability of bacteria (89.41–96.32%), that passed meaningfully through the simulated gastrointestinal tract (4.39–5.97 log reduction), largely maintaining their probiotic properties, being a promising environmentally-friendly carrier for probiotic LGG.

## 1. Introduction

Probiotic bacteria (defined as: “living microorganisms which exert beneficial effect on the host health when consumed in adequate amounts”) are intensively studied worldwide [1]. *Lacticaseibacillus rhamnosus* GG (LGG) is a Gram-positive Lactic Acid Bacterium (LAB), and one of the best-studied probiotics in clinical trials. This strain exhibits most of the features required for probiotics, and has been shown to be safe and non-pathogenic [1,2,3]. It has been previously reported that LGG exerts beneficial effects in treating and/or preventing several disorders, including ulcerative colitis, diarrhea, atopic dermatitis, rotavirus infections, sepsis and meningitis [2,3,4,5]. The competition with pathogens for binding sites and production of antimicrobial compounds are the main mechanisms suggested to contribute to the probiotic action of LGG in the gastrointestinal tract (GIT) [2,3,4,6]. One of the LGG characteristics responsible for its health-benefit properties is resisting low pH levels, the ability to adhere mucus and epithelial cells as well as prolonged residence in GIT [1,3,4]. Probiotic microorganisms should be present in the product at minimum numbers of 10^6^–10^7^ CFU (Colony Forming Units) per mL or g of product [7,8]. The technological issues related to the development of products containing beneficial microflora at recommended levels, maintaining their viability during shelf life and stabilization throughout the GIT are a challenge [9].

Spray drying is a fast and cost-effective technique to produce powders (solid microparticles) from starting liquid raw materials, being one of the promising processes to produce dry, probiotic formulations, as well as a strategy to protect and improve their viability within the GIT [9,10,11,12,13,14]. Moreover, spray drying is one of the encapsulation methods, which refers to a process where the active ingredients or cells are surrounded (encapsulated) by a protective continuous film of polymeric materials [15]. Polysaccharides and proteins are widely used to prepare carriers/delivery systems, playing a pivotal role in their structure and stability [16,17,18]. Many natural-based wall/carrier materials have been proposed for improving LAB (including probiotic strains) survivability during spray drying and passage through the GIT [18,19,20]. Various carriers such as skim milk and calcium-fortified skim milk [11,21], whey proteins [22], maltodextrin [1], native rice starch and inulin [23], fructooligosaccharides [14], agava fructans and buttermilk proteins [24], trehalose [25], polysaccharides (alginate, carrageenan, pectin, xantan, gellan) [1], vegetable juices [26] as well as almond milk [10] can be considered a promising strategy to improve stability and viability of probiotics. However, there are very limited information about maintaining probiotic properties of LAB after spray drying [27]. An innovative idea is to obtain reconstituted plant water extracts (also known as plant milks) and was already reported in for spray-dried reconstituted soymilk and almond-based milk [10,28]. Reconstitution (as a result of rehydration by adding water) has been known for decades in the case of spray-dried animal (bovine, camel) milks [29]. Moreover, due to the increase in vegan, vegetarian and allergic consumers, food and pharmaceutical industries are currently searching for alternatives in plant-based carriers. Indeed, plant-based products are raising interest as innovative potential carriers ensuring probiotics for vegan consumers [8,30,31,32]. Therefore, sourcing biopolymers such as plant-derived proteins and polysaccharides is of great importance for the development of innovative functional products, such as new probiotic carrier matrices [16,18,33].

Plant by-products (such as pomace, seeds, kernels, stalks, oilseed cakes and meals) are produced in large quantities worldwide, and have great potential to be used to provide phytochemicals and high value-added biopolymers [18]. The use of new types of biopolymers is important to the green label trend—the term green refers to origin, which involves waste and by-products valorization [12,18]. Oil cake/oil meal is a by-product of the extraction of oil from seeds [34,35]. These residues are an abundant source of compounds (antioxidants and biopolymers such as proteins, polysaccharides, fibers) with beneficial properties that can be used in many fields. Another advantage is the economic aspect: oil cakes are a cheap, safe material available all year round. The use of oil cakes can be a sustainable alternative to reduce waste and also contributes to the development of new low-cost products [34]. Flaxseed oil cake (FOC) is an inexpensive by-product of pressing flaxseed (*Linum usitatissimum* L.) oil and is a source of many bioactive substances such as proteins, polysaccharides, fiber and polyphenols. Flaxseed global production is estimated to be more than 1.2 million tons [36], thus generating large amounts of FOC. The valorization of FOCE (flaxseed oil cake extract) is a relatively new issue. FOCE is a liquid mixture obtained by hot extraction from FOC, the main components of which are primarily protein (FP—flaxseed protein) and mucilage (also known, as flaxseed gum—FG), abundant also in antioxidants [13,35,37]. Some several technological applications of FOC and FOCE are reported, including spray-dried emulsions and powders with emulsifying activity [12,13,37], and as dairy alternatives [38,39,40] which demonstrates the potential of this valuable agro-industrial by-product in the concept of circular economy and sustainable development. A feature of FG is the formation of a multiform structure and increase the resistance of multiphase systems to environmental stresses, thus FG can be used as a thickener, stabilizer, gelling agent and emulsifier [17,35,41]. Moreover, flaxseed mucilage showed good prebiotic potential, that can protect LAB cells from the adverse gastric environment and digestion [15,42]. In fact, the application of prebiotics (that could be used by bacteria for survival through the GIT) for encapsulation purposes has shown many advantages [23,43]. On the other hand, FP has good film-forming properties that can participate in encapsulation processes [44,45]. Previous works have shown that FOCE (used as liquid matrix or spray-dried powders) can be used as a stabilizing agent due to the synergistic effect of FP and FG [13,35,37]. Moreover, flaxseed polysaccharides have mucoadherent properties, and could be used in therapeutic or cosmetic applications [46]. Mucoadhesive polymers have been found to facilitate probiotic adhesion in the GIT, therefore are good candidates as encapsulating polymers for probiotic delivery systems [47]. Several authors reported the application of flaxseed mucilage as encapsulating material for LAB and their good survivability after spray drying and during incubation in simulated gastrointestinal conditions [42,48,49]. There are some reports indicating that the high efficiency of microencapsulation could be achieved by synergistic effect of the wall polymers (charged polysaccharides and amphiphilic proteins) through electrostatic interactions [15,17,20,21,41]. Therefore, FOCE presumably could act as an effective encapsulating agent and carrier for LAB, due to its biopolymers (proteins and polysaccharides) content.

The objective of this research was to valorize spray-dried FOCE as a natural carrier for probiotic LGG. Particularly, this study was designed to evaluate the influence of the spray drying process on powders properties as well as viability and probiotic properties of LGG in initial and reconstituted FOCE under simulated conditions of the GIT.

## 2. Materials and Methods

### 2.1. Materials and Chemicals

Flaxseed oil cake (FOC) was purchased from ACS Sp. z o.o. (Bydgoszcz, Poland). The proximate composition of FOC (based on supplier information) was: solids—80.50%, including: proteins—41.97%; carbohydrates—27.99%; fiber—6.29%; fat—6.11%; ash—4.50%. *Lacticaseibacillus rhamnosus* GG (ATCC53103), was procured from ATCC (Manassas, VA, USA). Buffered peptone water, microbiological agar, MRS agar and broth were purchased from Oxoid (Basingstoke, UK). Potassium chloride, sodium chloride, potassium thiocyanate, disodium hydrogen phosphate, monosodium dihydrogen orthophosphate, calcium chloride, sodium hydrogen carbonate, hydrochloric acid, ammonium chloride, sodium peroxide, urea, α-amylase, uric acid, mucin from porcine stomach (type II), glucose, glucuronic acid, glucosamine hydrochloride, bovine serum albumin (BSA), pepsin, pancreatin, oxgall, Triton X-100, cholesterol, ethanol, ninhydrin, glacial acetic acid, cadmium chloride and hexadecane were purchased from Merck Chemical (Saint Louis, MI, USA). All reagents were of analytical grade.

### 2.2. Preparation, Fermentation and Spray Drying of Flaxseed Oil Cake Extract with LGG (FOCE-LGG)

The preparation and fermentation of FOCE-LGG was carried out as described in previous study [40]. Briefly, FOC was extracted with hot distilled water (1:10 *w*/*w*, 90 °C, 1 h, 250 rpm), then cooled down to 20 °C, centrifuged (4000 rpm, 30 min) to obtain FOCE. Subsequently FOCE was filtered, homogenized (12000 rpm, SilentCrusherM, Heidolph, Germany), and fermented by LGG (42 °C, 24 h). Powdered FOCE-LGG samples were obtained using a laboratory scale spray dryer (Büchi B-290, Büchi Labortechnik AGT, Flawill, Switzerland). Three inlet temperatures were used: 110 °C, 140 °C and 170 °C. The outlet temperature was maintained at 55 ± 5 °C, and the air flow was 40 m^3^/h. The powders were collected in a sterile glass collection vessel. Total solids content (TSC) of non-dried FOCE and powders (denoted as FOCE-LGG-110, FOCE-LGG-140 and FOCE-LGG-170) was evaluated following the standard method (no. 968.11) of AOAC (Association of Official Agricultural Chemists) [50].

### 2.3. Powders Characterization

The powders were characterized for water activity (a_w_, MS1 Set-aw, Novasina, Lachen, Switzerland), morphology (SEM microscopy, Vega 3 LMU, Tescan, Brno, Czech Republic), chemical composition (FTIR spectroscopy, Perkin Elmer Spectrophotometer 100, Waltham, MA, USA), and particles size distribution (Mastersizer 2000 with a Scirocco 2000 dry sampling system, Malvern Instrument Ltd., Worcestershire, UK) as described in previous studies [12,13,37]. Moreover, bulk (ρ_b_) and tapped (ρ_t_) densities were determined as described elsewhere [13], using the following formulas:

(1) $$\rho_{b}=\frac{\mathrm{powder}\;\mathrm{weight}\;\left(g\right)}{\mathrm{powder}\;\mathrm{volume}\;\mathrm{after}\;\mathrm{tapping}\;\left({\mathrm{cm}}^{3}\right)}$$

(2) $$\rho_{t}=\frac{\mathrm{powder}\;\mathrm{weight}\;\left(g\right)}{\mathrm{powder}\;\mathrm{volume}\;\mathrm{after}\;\mathrm{tapping}\;\left({\mathrm{cm}}^{3}\right)}$$

The Carr’s index and Hausner ratio were used to express flowability and cohesiveness of the powders [13]. The indexes were calculated from the ρ_b_ and ρ_t_ values, based on the following equations:

(3) $$C\left(\%\right)=\frac{\rho_{t}-\rho_{b}}{\rho_{t}}\times 100$$

(4) $$\mathrm{HR}=\frac{\rho_{t}}{\rho_{b}}$$

Whereas for powders evaluation a scale based on European Pharmacopoeia standards (Table 1) was used [51].

### 2.4. FOCE-LGG Reconstitution, Determination of pH, Free Amino Acids, Sulfhydryl Groups (-SH) and Disulfide Bonds (-S-S-) Contents

The reconstitution of the FOCE-LGG samples was carried out by adding the individual powders to distilled water to obtain the initial dry matter content of the starting samples (taking into account the total solids content of the powders), and then stirred until a homogeneous (37 °C, 20 min, 50 rpm) [13]. pH measurements of the samples were carried out directly at 25 °C using a pH-meter (CP-411, Elmetron, Zabrze, Poland). Free amino acids were determined using ninhydrin-Cd reagent as described elsewhere [39]. The sulfydryl groups (-SH) and the disulfide bonds (-S-S-) contents were analyzed according to the protocol described by Gong et al. [52].

### 2.5. Enumeration of LGG Counts

The LGG counts in FOCE-LGG, reconstitution and at each stage of the GIT (mouth, stomach and small intestine) was determined in compliance with ISO 6887-1:2017. The samples (1 mL) were diluted with 9 mL of sterile buffered peptone water, and further ten-fold serial dilutions were prepared. LGG counts were determined on MRS agar after incubation at 37 °C under anaerobic conditions for 72 h. The enumeration of all microorganisms was performed in triplicate (by counting plates with 30–300 colonies) and the viable cell counts were expressed as log CFU/mL of the samples [40].

### 2.6. Gastrointestinal Tract Simulator (GITS)

The Simulator of Human Intestinal Microbial Ecosystem (SHIME) was adopted to assess the behavior of selected features of the LGG administered in the tested products. The model consisted of three bioreactors simulating the processes taking place in selected sections of the digestive system—mouth (Reactor 1), stomach (Reactor 2) and small intestine (Reactor 3), completed with the mucin adherence test (ascending colon). Control of filling and intake of ingredients at specified times was carried out using peristaltic pumps with a total retention time of 6 h. The dwell time in individual reactors did not exceed 2.5 h and was dependent on the volume [53,54]. The content of each bioreactor was magnetically stirred (33 rpm, IKA, Staufen, Germany) and maintained at a temperature of 37 °C ± 1 °C. Cultures were incubated in anaerobic conditions modified with the composition of N_2_ and CO_2_ [55]. The pH and temperature values were monitored electronically (Adwa, Szeged, Hungary).

#### 2.6.1. Simulated Digestion

##### Saliva Preparation

The saliva medium was prepared according to Oomen et al. [56] and consisted of: 10 mL of 8.96% KCl, 10 mL of 2.0% KSCN, 10 mL of 5.7% Na_2_PO_4_, 1.7 mL of 17.53% NaCl, 1.8 mL of 4.0% NaOH, 8 mL of 2.5% urea, 145 mg α-amylase, 15 mg uric acid and 50 mg mucin filled up to 500 mL with distilled water. The pH was adjusted to 7.0 ± 0.2 with 0.1 M NaOH.

##### Gastric Medium Preparation

Gastric medium was prepared based on protocol of Oomen et al. [56] with some modifications. The electrolyte solution consisted of: 15.7 mL of 17.53% NaCl, 3.0 mL 8.88% NaH_2_PO_4_, 9.2 mL of 8.96% KCl, 18 mL of 2.22% CaCl_2_, 10 mL of 3.06% NH_4_Cl, 10 mL of 6.5% glucose, 10 mL of 0.2% glucuronic acid, 3.4 mL of 2.5% urea, 10 mL of 3.3% glucosamine hydrochloride and organic solution: 1.0 g BSA, 1.0 g pepsin, 3.0 g mucin. The mixture was filled up with 500 mL distilled water. The pH value of the gastric juice was adjusted to 3.0 ± 0.2 by 0.1 M HCl.

##### Intestinal Juice Preparation

Intestinal juice was prepared as described by Bondue et al. [57] by mixing in 0.9 g of pancreatin, 4.0 g of ox-gall, 2.5 g NaHCO_3_ and filled the mixture to 1000 mL with distilled water. Sodium hydroxide solution (0.1 M) was used to adjust to pH 6.2 ± 0.5.

##### Mucin Agar Preparation

Mucin agar was prepared by boiling distilled water and 1% agar, when the mixture was cooled to 65 °C, 5% mucin from porcine stomach (type II) was added. The pH was adjusted to 6.8 with 0.1 M NaOH [58,59].

#### 2.6.2. Characterization of Survival in GITS

The samples were mixed with saliva solution in a ratio of 10:1 (*v*/*v*). Incubation was carried out for 10 min with constant stirring (50 rpm) (Reactor 1). Subsequently, 50 mL of mixtures (2 mL/min) were transferred to the prepared gastric medium (Reactor 2), to obtain the final ratio 5:8 (*v*/*v*), and pH 3.0 ± 0.2 (maintained by using 0.1 M HCl). This mixture was incubated for 2 h, then transferred (2.0 mL/min) to the next Reactor 3 (intestinal juice). The volume transfer was calculated to give a final ratio of 5:6 (*v*/*v*). The pH was adjusted with 0.1 M NaOH, followed by incubation for another 2 h. The final step in controlling LGG in GITS was to evaluate the ability to colonize mucin as the active layer of the gut. For this purpose, 1 mL of the mixture was taken from Reactor 3 and added to a 12-well plate covered with 1.2 mL of mucin agar. Incubation was carried out at 37 °C with constant agitation (30 rpm). After 80 min, unadhered bacteria were removed by rinsing three times with PBS. LGG cells remaining on mucin agar were separated from the medium using 0.5% Triton X-100 in PBS. The total number of adhered cells (in triplicate) was evaluated by the plate method described above. The obtained bacterial counts were converted in accordance to counts resulted from Reactor 3.

The survival of bacteria (SUR) LGG in GITS (for saliva, gastric juice and intestinal juice) was calculated according to Sumeri et al. [54]:

(5) $$\mathrm{SUR}=\frac{{\mathrm{CFU}}_{t\;}\times {\;V}_{t}}{{\mathrm{CFU}}_{F\;}\times {\;V}_{F}}$$

where: CFU_t_ stands for instantaneous concentration of bacteria (CFU/mL), V_t_ is the volume of culture (mL) in the bioreactor vessel at time point t, CFU_F_ is the concentration of bacteria in FOCE-LGG variants (CFU/mL), and V_F_ is the volume of FOCE-LGG variants (mL) injected into the vessel.

### 2.7. Determination of Probiotic Properties of LGG on GITS Stages

#### 2.7.1. Cholesterol Binding Activity

Cholesterol binding (CH_b_) activity was determined on the basis of its loss after incubation with FOCE-LGG and FOCE-LGG-110, FOCE-LGG -140 and FOCE-LGG -170 samples obtained after passage through SHIME. Samples taken from Reactor 3 as well as from “fresh” FOCE-LGG were loaded into 12-well plates with 1% cholesterol (dissolved in 96% ethanol) to obtain a final cholesterol concentration of 1.66 g/L. The samples were incubated for 18 h (30 °C), then centrifuged (Centrifuge MPW 351R, 7500 rpm, 10 min, 4 °C) to separate the culture fluid. In order to determine the cholesterol residues in the samples, the procedure of Cholesterol RTU kit (BioMerieux, Marcy l’Etoile, France) was followed. The absorbance measurement was performed using the NanoDrop ND 1000 spectrophotometer at 500 nm. The percentage of bound cholesterol from the environment was calculated according to the formula:

(6) $${\mathrm{CH}}_{b}=\frac{\left[\left(A-B\right)\times 100\right]}{A}$$

where: A is the initial absorbance of mixture, B is the absorbance of mixture after inxubation.

#### 2.7.2. Surface Hydrophobicity Assay

The BATH (Bacterial Adhesion to Hydrocarbons) method with hexadecane was used to assess hydrophobicity of LGG cells following procedure of Rahman et al. [60] with some changes. Five milliliters of bacterial suspensions were mixed with 2 mL of hexadecane by vortexing for 60 s, then incubated for 2 h (37 °C). Changes of absorbance were measured at 620 nm using a spectrophotometer (BioPhotometer D30, Eppendorf). The hydrophobicity (SH%) was expressed according to formula:

(7) $$\mathrm{SH}\%=\left[\frac{A_{0}-A}{A_{0}}\right]\times 100$$

where: A_0_ is the initial OD at 620 nm and A is the final OD at 620 nm.

It was assumed that result: SH > 70% indicates high hydrophobicity; SH 20–70% indicates medium hydrophobicity, whereas SH < 20% indicates low hydrophobicity.

### 2.8. Statistical Analysis

All experiments were carried out three times. Results are expressed as mean ± standard deviation. One-way and two-way ANOVA with Tukey’s tests were conducted using the Statistica 13.0 software (StatSoft, Kraków, Poland), and *p* values < 0.05 are considered to be statistically significant.

## 3. Results and Discussion

### 3.1. Physicochemical Properties of Powders

It was noticed that total solids content (TSC) of FOCE was 3.11%, whereas TSC of FOCE-LGG-110, FOCE-LGG-140 and FOCE-LGG-170 powders was 89.01%, 90.81% and 91.13%, respectively (Table 2). Based on those results, the adequate amounts of distilled water necessary for reconstitution (to obtain initial TSC) were calculated. Moisture content between 4–10% in spray-dried powders was considered a value for this parameter associated with good quality [61,62]. The spray drying temperature significantly influenced the water activity, and values in a range 0.453 ± 0.002 (FOC-LGG-110) and 0.297 ± 0.005 (FOCE-LGG-170) were noticed (*p* < 0.05). These results are slightly higher than reported by Bustamante et al. [49]. However similar a_w_ values were reported in previous studies for FOCE spray-dried powders [12], and FOCE-based spray-dried emulsions [37].

**Figure 1:**
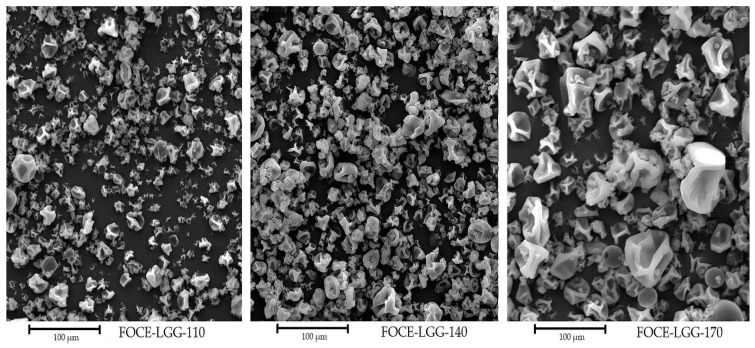
Morphology of FOCE-LGG spray-dried powders.

The morphology of the spray-dried powders is illustrated in Figure 1, whereas particles size, bulk and tapped densities, flowability, cohesiveness, and water activity are summarized in Table 2. As can be seen, the powders particles were relatively spherical. Moreover, some folds and wrinkles with dentures were noticed. Spray drying generally causes the powder particles to shrink due to water evaporation [63]. For the powder produced at 170 °C the most uneven particle size morphology was noticed, attributed to the highest temperature. A similar morphology of spray-dried FOCE powders applied as emulsifying agents were observed in previous study [12]. Rajam and Anandharamakrishnan also reported spherical morphology of fructooligosaccharide-based microcapsules with *Lactobacillus plantarum* [64]. No bacterial cells were visible on the particles surface, which indicates a good encapsulation of the LGG strain. This observation is consistent with results of Bustamante et al. who reported that *L. plantarum* and *Bifidobacterium infantis* cells were not visible on the surface of the spray-dried flaxseed mucilage powders [49]. Powder particle size is an important quality parameter (the size may determine the degree of cell protection) and must be suitable for encapsulating probiotics, which typically exhibit relatively large sizes in the range of 1 to 5 µm (LGG has average size between 1 and 2 µm) [1,48]. The highest average particles size (42.91 ± 0.03 µm) was observed for powder FOCE-LGG-170, whereas the lowest was noticed for powder FOCE-LGG-110 (32.91 ± 0.05 µm). These results are comparable with reported for FOC-based spray-dried emulsions [37], and are in line with results reported by other authors using flaxseed components for encapsulation [42,48]. On the other hand, they are higher than reported by Rajam and Anandharamakrishan using fructooligosaccharide for *L. plantarum* encapsulation [64]. With increasing temperature a hard biopolymeric layer formation is intensified, which leads to particle inflation, increasing the particle size [12,33].

Morphology directly influences characteristics of encapsulated powders such as bulk and tapped densities as well as flowability and cohesiveness [64]. As presented in Table 2 the spray drying temperature significantly influenced ρ_b_ and ρ_t_ of the powders (*p* < 0.05). The highest ρ_t_ (0.37 ± 0.01 g/cm^3^) was observed for powder FOCE-LGG-110. On the contrary, the lowest was noticed for sample FOCE-LGG-170 (0.34 ± 0.02 g/cm^3^). Similarly, for this powder the lowest ρ_b_ was found (0.27 ± 0.01 g/cm^3^). Those results are lower than reported for fructooligosaccharide-based microcapsules [64], and for FOCE-based spray-dried emulsions [13]. As the inlet temperature increases, the water in the feed fluid evaporates faster and a crust is formed on the surface of the particles, generating water vapor inside the particles, thus the high pressure generated in the particles can cause the release of water vapor from the pores which leads to a decrease in bulk density [65]. However, it should be pointed out that the generated pressure can be crucial factor influencing bacteria survivability. CI in the range 0–10 and HR between 1 and 1.10 are values indicating good flowability [51,63]. In our study the lowest HR (1.09 ± 0.01) and CI (8.57 ± 0.21%) were noticed for sample FOCE-LGG-110. Based on a scale presented in Table 1 this powder could be classified as excellent, thus indicating its good potential for storage and handling. With increasing temperature a tendency to HR and CI to increase was observed (*p* < 0.05), thus powders FOCE-LGG-140 and FOCE-LGG-170 could be classified as possible. The results observed for powders dried at 140 °C and 170 °C are comparable with those reported for spray-dried camel milk powder [63] and FOCE-based emulsions [13].

**Figure 2:**
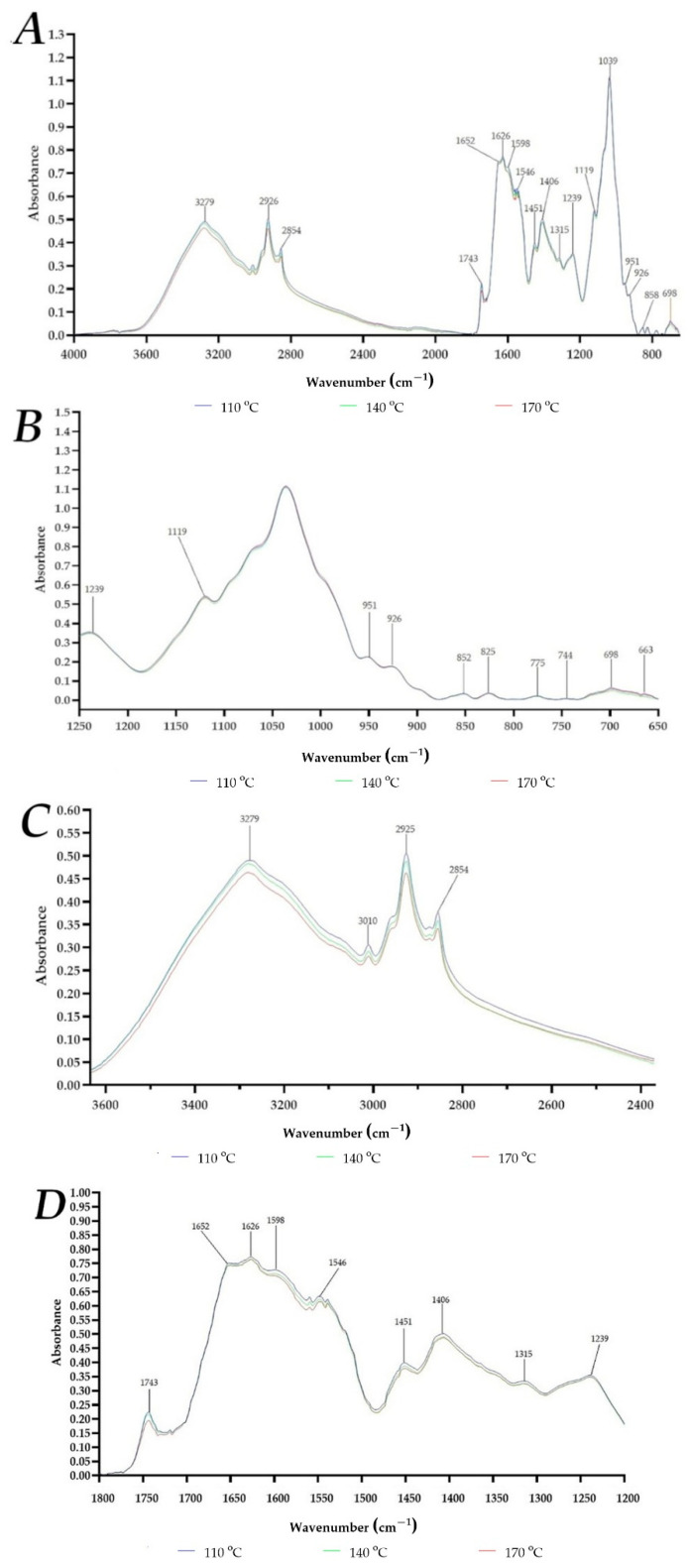
FTIR spectra of FOCE-LGG powders. (**A**) whole FTIR spectrum (4000–650 cm^−1^); (**B**) in the range of 1250–650 cm^−1^ (polysaccharides); (**C**) in the range of 3600–2400 cm^−1^ (−OH, −NH, −CH2 and −CH3 groups); (**D**) in the range of 1800–1200 cm^−1^ (proteins).

The FTIR spectra of the powders are presented in Figure 2. The characteristic CH_3_ and CH_2_ stretching bands at approximately 2926 cm^−1^ and 2854 cm^−1^, respectively, can be seen [12,44]. A band at 3279 cm^−1^ can be attributed to N-H stretching vibrations of the primary amide structure as well as inter-and intra-molecular OH groups, C-H stretches, and residual water [12,44]. At approximately 1652 cm^−1^, 1626 cm^−1^, 1598 cm^−1^ and 1546 cm^−1^ a strong amide band was noticed which can be imputed to C=O stretching vibrations, N–H stretching vibrations, N–H bending vibrations, and C–N bending vibrations in proteins [12,33,52]. A decrease of those bands intensity with increasing temperature can be linked with water removal by evaporation, presumably partial denaturation and hydrolysis of FP as well as bacterial proteins [12,33,52]. In fact, the denaturation of FP during spray drying was reported in previous studies [12,42]. Bands detected at 1039 cm^−1^ (angular deformation of =CH and =CH_2_ bonds), 858 cm^−1^ (deformation of C_1_-H and CH_2_) and 698 cm^−1^ (C-O-C pyranose ring stretching) are assignable to mucilage polysaccharides, and no intensity changes were observed suggesting FG stability during spray drying. Interestingly, a band at 1743 cm^−1^, and its intensity decrease with increasing temperature was observed. This signal, characteristic of various carbonyl groups such as aldehydes, ketones and carboxylic acids, was imputed in previous study to a small content of flaxseed oil residues in FOCE dried at room temperature [35]. However in another study, for FOCE spray-dried (at 160 °C, 180 °C and 200 °C) powders this signal was detected only for sample dried at 200 °C, and was imputed to the formation of hydrogen bonds between C=O and N-H groups of proteins with O-H groups of polyphenols and polysaccharides [12]. In fact, fermentation with LGG significantly increased polyphenolics and flavonoids content of FOCE, due to enzymatic activity and delinking of bounded polyphenolic compounds from proteins and cell walls, as reported in previous study [40]. Moreover, several authors reported that high efficiency of encapsulation of bacteria could be also achieved by synergistic effect of flaxseed components and other wall polymers due to electrostatic interactions and formation of hydrogen bonds with other wall polymers [15,17,33]. It should be emphasized that bacterial cell surfaces have a net negative electrostatic charge due to the presence of phosphoryl and carboxyl groups on the macromolecules of the outer cell envelope that are exposed to the extracellular environment [66]. Therefore, it is reasonably to suggest that in fermented FOCE formation of some survival-enhancing interactions between bacteria and/or matrix biopolymers can occur. Nevertheless, further in-depth analysis is still required to understand this complex mechanism of action.

### 3.2. LGG Survivability after Spray Drying, FOCE Acidity, Free Amino Acids, Sulfydryl Groups and Disulfide Bonds Contents

The LGG counts in FOCE and reconstituted samples, survivability rate, pH, free amino acids, sulfydryl groups and disulfide bonds contents changes are listed in Table 3. A statistically significant increase of pH was noticed, presumably attributed to lactic acid degradation during spray drying (*p* < 0.05). It was observed that spray drying did not reduce significantly viable LGG counts in FOCE-LGG samples after reconstitution (*p* > 0.05). The high processing temperature and low moisture can reduce bacterial viability due to many factors, including cell membrane dehydration (disrupting its permeability) as well as damage to cellular components [49].

The highest LGG survivability rate was observed for sample FOCE-LGG-110 (96.32%), followed by FOCE-LGG -140 (95.88%) and FOCE-LGG-170 (89.41%). A similar survivability of *L. plantarum* (ATCC8014) was reported by Lipan et al. for almond milk spray-dried at 170 °C, 180 °C and 190 °C [10]. This viability is also comparable with results reported by other authors for encapsulated spray-dried *Lactobacillus* strains [42,67], and higher than reported by Ananta et al. [62] as well as by Akanny et al. [1]. The high survivability of LGG in spray-dried FOCE can be presumably attributed to its two main fractions: flaxseed gum (FG) and flaxseed protein (FP), having high stabilizing activity [35]. In previous studies we demonstrated that FOCE can be spray-dried and form good protective coatings [13,37]. Other authors also reported protective role of proteins such as casein, as well as whey and buttermilk proteins during spray drying of dairy products [24,68]. Moreover, carbohydrates play also pivotal role as thermoprotectants [10,25,68,69].

It is suggested that the protective effect of small carbohydrates and protein molecules (such as trehalose, mannitol, maltodextrin, lactose, casein) is due to their ability to replace water in bacterial membranes, thus maintaining their structural and functional integrity [1]. However, steric hindrances of high molecular weight polymers due to their large size prevent them from interacting with dried membrane proteins and lipids beneath the bacterial cell wall. Therefore, the complete maintenance of the structural and functional integrity of the bacterial cell membrane during the spray drying process may be disrupted [43]. FP and FG are high molecular weight polymers [17,70,71] and therefore most likely interact directly with polar groups of cell wall peptidoglycan, lipoteichoic acids and teichoic acids, thus providing stability to bacterial cells [1,71]. Presumably, high water holding activity of FOCE (due to FG content) was also an important factor for LGG survivability [35].

It is known that spray drying induces thermal denaturation of proteins and their partial degradation, changing their native structure, resulting in change protein–protein hydrophobic, electrostatic, hydrogen-bonding and disulfide–sulfhydryl interactions [12,48,52,72]. Exposure of sulfhydryl and hydrophobic groups (due to denaturation of proteins by spray drying) can reduce the swelling rate of microcapsules and diffusion of cells, which in turn reduces the lethal effect on bacteria [42,63,65]. In fact, partial denaturation of FOC proteins (resulting in changes of sulfhydryl groups and disulfide bonds) as a result of spray drying was reported [41]. Indeed, in this study a significant decrease (approximately two-fold) of sulfhydryl groups and increase of disulfide bonds in powders in comparison with FOCE was observed (*p* < 0.05). The elevated temperature caused a similar trend when only powdered samples were considered (*p* < 0.05). However, these changes could be also presumably partially attributed to denaturation of bacterial proteins, indicating the lethal effect of temperature [42,49]. Moreover, the content of free amino acids significantly increased in spray-dried powders indicating partial hydrolysis of FP (*p* < 0.05). The major fraction of FP is globulin, which shows poor mechanical properties because most of the hydrophobic and -SH groups are hidden inside the protein molecules [42]. According to Bustamante et al. the protein-protein interactions, disulfide crosslinking and hydrogen bonding make the denatured FP proteins stiffer, stronger and stretchable [42]. Although denaturation of matrix proteins may affect bacterial survival, the aforementioned generation of high pressure could have contributed to the fact that with increasing temperature, bacterial survival decreased. Nevertheless, the high survival of bacteria at 110 °C and 140 °C indicates, that, to some extent protein denaturation could play a significant role in enhancing their survival.

### 3.3. LGG Survivability in Saliva Juice, Gastric Juice and Intestinal Juice

The objective of the tests was to evaluate the influence of GIT stressing factors on the survival of LGG introduced in the biopolymeric matrix. The decrease of viable LGG counts in FOCE and reconstituted samples in GITS parts (saliva juice, gastric juice, intestinal juice) are listed in Table 4. The statistical comparison revealed no influence of the spray drying temperature on the sensitivity of LGG strains in GITS. In all of the analyzed cases it was found that a critical factor influencing the survival of bacteria is low pH (2.5). During 120 min of exposure to gastric juice, the number of LGG counts decreased by 4 log from the initial values (*p* < 0.05). Those results share a number of similarities with findings of Ritter et al. [73], who suggested that the greatest reduction in cell number occurs within the first 30 min of gastric juice and bile salts administration, reaching as much as 5 log less after 2 h. Simulation of GI transport to the small intestine did not show such a significant lethal effect on LGG (*p* < 0.05). The observed decrease in the number of bacteria by <0.63 log can be considered as an increase in the environmental stress that occurs in a short time for bacteria. Neutralization of the environment to pH 6.2 ± 0.5 and the introduction of ingredients simulating the composition of intestinal juice limited the physiological regenerative and adaptive abilities of bacteria. As can be seen, LGG survival in fermented products showed that the stress behavior pattern of bacteria in individual GI fragments is similar. However, the analysis carried out showed that the reduction in the number of bacteria in the FOCE-LGG (not subjected to the spray drying process) is at least 1 log lower (*p* < 0.05). The results share a number of similarities with bacterial strains levels after passage through GITS reported in the literature [1]. Taking the survival rate in GITS as the basic indicator of the probiotic potential of LGG strains it should be considered that the tested products can be used as carriers for probiotic strains. According to Ranadheera et al. [7], some probiotic carriers of plant origin, can provide a protective environment during digestion and reduce probiotics exposure to harsh conditions in the GIT. In addition, those carriers may contain various protective components such as prebiotics, that interact with probiotics in the terminal part of the GIT and stimulate both their growth and activity [7]. In addition to direct prebiotic activity, many plant oligosaccharides and polysaccharides assist in the delivery of probiotics to target sites [7,23,69]. Prebiotic effects of FG have also been described [17,74].

### 3.4. Maintenance of LGG Probiotic Properties—Hydrophobicity, Adhesion to Mucin and Cholesterol Reduction

The hydrophobicity of LGG in FOCE and in reconstituted samples after passage through GITS is listed in Table 5. Based on hydrophobicity scale [60], it was found that the cellular LGG in the tested products, after passing through GITS, is characterized by a medium level of hydrophobicity. The obtained SH% values ranged from 25.77 ± 4.69% to 28.95 ± 8.74% and were not statistically significant (*p* > 0.05). Such similar results are the result of using a specific strain of LGG in the fermentation process and maintaining the standard conditions in GITS. To exert beneficial effects, a probiotic must survive digestion in the GIT and adhere to the intestinal epithelium, which depends on the degree of hydrophobic properties of the microorganisms [10]. In general, hydrophobicity is a complex process dependent on environmental pH and induced by bile salts [70]. It is widely recognized that it influences the adhesion capacity to epithelial cells and thus offers potential benefits to bacteria colonizing the gastrointestinal tract.

**Figure 3:**
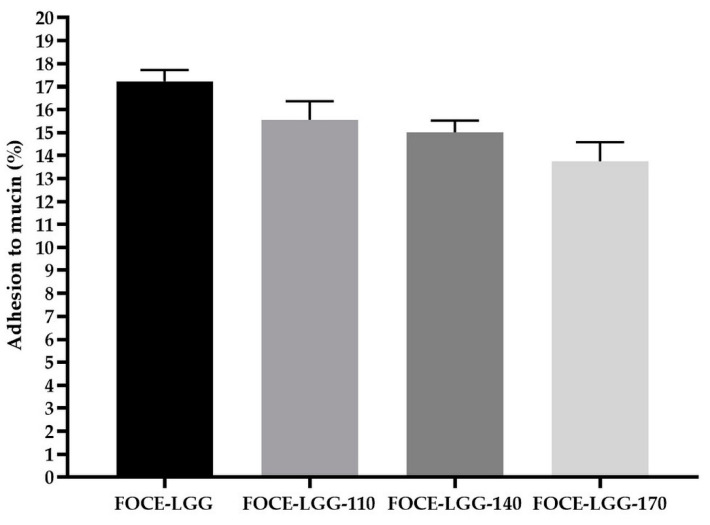
LGG adhesion to mucin.

The potential of LGG for adhesion to mucin is presented in Figure 3. The adhering capacity of LGG to mucin was significantly different (*p* < 0.05), however, is comparable. The determined levels of adherence obtained after incubation (13.74 ± 2.84% and 17.23 ± 3.50%, for FOCE-LGG-170 and FOCE-LGG samples, respectively) are consistent with the reports of van den Abbeele et al. [71] as well as Marzorati et al. [72]. Generally, adhesion is a process involving nonspecific (hydrophobic) and specific (ligand-receptor) interactions between the surface of bacterial cells and host cells. Cell wall specificity, including in particular: fimbriae, adhesins, exopolysaccharides, and surface layer and mucus binding proteins plays a pivotal role in maintaining an advantage in epithelial colonization. Golowczyc et al. reported that spray drying may influence some surface structures of *Lactobacillus* sp. used for adhesion, although no damage in the cell membranes were detected [27]. The simulations to mimic these in vitro processes are performed by various methods. In addition to using cell lines a mucin adherence test is widely used [75]. Mucin is the main component of mucus and the ability to adhere to it may determine long-term colonization by probiotic bacteria.

The results of cholesterol reduction are presented in Table 6. As can be seen, slightly lower values of cholesterol reducing ability were observed for reconstituted FOCE-LGG samples (*p* < 0.05). The lowest CH_b_ was noticed for sample FOCE-LGG-170 (20.73 ± 0.58%) and was approximately 10.65% lower when compared with FOCE-LGG (23.21 ± 0.65%). This result can be presumably attributed to observed reduction of viable LGG counts. Yadav et al. [76] found that dead cells also removed cholesterol from the broth, but the cholesterol removal capacity was lower compared to that found in the presence of live cells. On the other hand, many reports established the cholesterol lowering effect of probiotics up to a significant level as much as 22% to 33% [75] which is comparable to results obtained in present study. The bile salt hydrolase (BSH) activity was found to be responsible for the cholesterol-lowering effects of probiotic microbes [76]. However, other mechanisms such as cholesterol binding to bile acids and inhibition of micelle formation combined with the effect of fermentation on the production of short-chain fatty acids (SCFA) have been proposed as explanations for the potential cholesterol-lowering effects of *Lactobacillus* strains [77]. For all the samples a significant decrease of cholesterol reduction ability was noticed after passage through GIT (*p* < 0.05) and the lowest was observed for sample FOCE-LGG-170 (5.26 ± 0.45%). The influence of the pH of the environment is directly related to the activity of the BSH enzyme through which it is possible to partially co-precipitate cholesterol with bile acids and remove it from the organism. The target effect, however, depends on the strain used for the research, and in the case of *L. rhamnosus* NBHK007, a higher reduction of cholesterol was observed at pH 3.8–4.3 [78]. In addition, the presence of bile salts may have a limiting effect on the mechanisms determining the level of cholesterol reduction [78]. In the obtained effects, the correlation between the number of physiologically active cells and the obtained cholesterol reduction effect might be then considered.

## 4. Conclusions

Taking into account the increasing vegan/vegetarian and application of new “green” types of biopolymers an approach was applied in this work to use valuable industrial by-product as a source of biopolymers-abundant extract for spray drying and carrying of probiotic bacteria. The findings of our study confirmed that FOCE has a good potential to be considered as an encapsulating agent and natural carrier for probiotic *Lacticaseibacillus rhamnosus* GG. Due to biopolymers (flaxseed proteins and polysaccharides) content FOCE provided high survivability of LGG cells, that passed meaningfully through the simulated gastrointestinal tract, largely preserved their probiotic properties. However, further in vivo studies, including human clinical trials of matrix combinations and doses in different populations, are needed to confirm the health benefits of LGG in FOCE. In addition, further in-depth studies to demonstrate the effect of spray drying and reconstitution on the physicochemical properties, bioactivity and matrix biopolymers interactions of FOCE should be carried out.

## Figures and Tables

**Table 1:** Specifications for Carr’s index and Hausner ratio [51].

	Carr’s Index	Hausner Ratio
Excellent	0–10%	1.00–1.11
Good	10–15%	1.12–1.18
Fair	16–20%	1.19–1.25
Possible	21–25%	1.26–1.34
Poor	26–31%	1.35–1.45
Very poor	32–37%	1.46–1.59
Very very poor	>38%	>1.60

**Table 2:** Total solids content (TSC), particles size (D_4;3_), water activity (a_w_), bulk denisity (ρ_b_), tapped denisty (ρ_t_), Hausner Ratio (HR), and Carr’s Index (CI) of FOCE samples.

Sample	TSC (%)	D_4;3_ (µm)	a_w_	ρ_b_ (g/cm^3^)	ρ_t_ (g/cm^3^)	HR	CI (%)
FOCE	3.11 ± 0.05 ^d^	-	-	-	-	-	-
FOCE-LGG-110	89.01 ± 0.01 ^c^	32.91 ± 0.05 ^a^	0.453 ± 0.002 ^a^	0.32 ± 0.01 ^a^	0.37 ± 0.01 ^a^	1.09 ± 0.01 ^c^	8.57 ± 0.21 ^c^
FOCE-LGG-140	90.81 ± 0.03 ^b^	35.22 ± 0.18 ^b^	0.307 ± 0.001 ^b^	0.29 ± 0.02 ^b^	0.35 ± 0.01 ^b^	1.26 ± 0.05 ^a^	20.05 ± 0.16 ^b^
FOCE-LGG-170	91.13 ± 0.10 ^a^	42.91 ± 0.03 ^c^	0.297 ± 0.005 ^c^	0.27 ± 0.01 ^c^	0.34 ± 0.02 ^c^	1.27 ± 0.03 ^b^	21.62 ± 0.15 ^a^

Values are means ± standard deviation of triplicate determinations. Means with different letters in the same column are significantly different at *p <* 0.05.

**Table 3:** LGG counts, survivability rate, pH, free amino acids (FAA), sulfhydrul groups (-SH) and disulfide bonds (-S-S-) contents of FOCE-LGG samples.

Sample	LGG Counts (log CFU/mL)	Survivability Rate (%)	pH	FAA (mg Gly/mL)	-SH (µmol/g)	-S-S- (µmol/g)
FOCE-LGG	8.97 ± 0.50 ^a^	-	4.22 ± 0.01 ^d^	6.48 ± 0.11 ^a^	41.11 ± 0.33 ^a^	15.92 ± 0.17 ^d^
FOCE-LGG-110	8.64 ± 0.32 ^a^	96.32	4.36 ± 0.01 ^c^	8.91 ± 0.23 ^b^	98.48 ± 0.92 ^a^	27.50 ± 0.22 ^c^
FOCE-LGG-140	8.60 ± 0.27 ^a^	95.88	4.39 ± 0.01 ^b^	9.37 ± 0.17 ^c^	95.33 ± 0.56 ^b^	29.03 ± 0.15 ^b^
FOCE-LGG-170	8.02 ± 3.41 ^a^	89.41	4.43 ± 0.01 ^a^	10.02 ± 0.14 ^d^	93.25 ± 0.33 ^c^	29.87 ± 0.05 ^a^

Values are means ± standard deviation of triplicate determinations. Means with different letters in the same column are significantly different at *p <* 0.05.

**Table 4:** LGG survivability (decrease of viable counts) in saliva juice, gastric juice and intestinal juice.

		FOCE-LGG	FOCE-LGG-110	FOCE-LGG-140	FOCE-LGG-170
log CFU/mL					
SUR_sj_	start	−0.002 ± 0.41 ^Aa^	−0.04 ± 0.37 ^Aa^	−0.20 ± 0.52 ^ABa^	−0.02 ± 0.56 ^Aa^
	finish	−0.14 ± 0.12 ^ABa^	−0.11 ± 0.82 ^Aa^	−0.62 ± 0.36 ^ABa^	−0.40 ± 1.41 ^ABa^
SUR_gj_	start	−0.76 ± 0.71 ^ABa^	−1.35 ± 0.34 ^Ba^	−1.08 ± 0.48 ^ABa^	−1.20 ± 0.14 ^ABa^
	finish	−3.50 ± 0.53 ^Ab^	−4.12 ± 0.15 ^ABb^	−4.18 ± 0.24 ^ABb^	−4.28 ± 2.61 ^ABb^
SOR_ij_	start	−4.21 ± 0.36 ^Aa^	−5.48 ± 0.35 ^Ba^	−5.35 ± 0.65 ^ABa^	−5.16 ± 0.71 ^Ba^
	finish	−4.39 ± 0.19 ^ADb^	−5.83 ± 0.12 ^Bb^	−5.97 ±0.89 ^BDb^	−5.42 ± 0.14 ^BCDb^

SUR_sj_—survival in saliva juice, SUR_gj_—survival in gastric juice, SOR_ij_—survival in intestinal juice; Values are means ± standard deviation of triplicate determinations. Means with different lowercase in the same column are significantly different at *p* < 0.05. Means with different uppercase in the same row are significantly different at *p* < 0.05.

**Table 5:** Surface hydrophobicity of FOCE-LGG samples.

Sample	SH%
FOCE-LGG	25.77 ± 4.69 ^a^
FOCE-LGG-110	28.95 ± 8.74 ^a^
FOCE-LGG-140	26.75 ± 5.87 ^a^
FOCE-LGG-170	27.56 ± 8.12 ^a^

Values are means ± standard deviation of triplicate determinations. Means with different letters are significantly different at *p <* 0.05.

**Table 6:** Cholesterol biding ability (%) of FOCE-LGG samples before and after simulated passage through SHIME.

	FOCE-LGG	FOCE-LGG-110	FOCE-LGG-140	FOCE-LGG-170
CH_b_—before SHIME	23.21 ± 0.65 ^Aa^	22.05 ± 0.72 ^ABa^	21.84 ± 1.12 ^Ba^	20.73 ± 0.58 ^Ba^
CH_b_—after SHIME	8.17 ± 0.70 ^Ab^	7.94 ± 0.98 ^Ab^	7.20 ± 0.65 ^Ab^	5.26 ± 0.4 ^Bb^

Values are means ± standard deviation of triplicate determinations. Means with different lowercase in the same column are significantly different at *p <* 0.05. Means with different uppercase in the same raw are significantly different at *p <* 0.05.

## Data Availability

The data presented in this study are available on request from the corresponding authors.

## References

[1] Akanny E., Bourgeois S., Bonhommé A., Commun C., Doleans-Jordheim A., Bessueille F., Bordes C. (2020). Development of enteric polymer-based microspheres by spray-drying for colonic delivery of *Lactobacillus rhamnosus* GG. Int. J. Pharm..

[2] Westerik N., Kort R., Sybesma W., Reid G. (2018). *Lactobacillus rhamnosus* probiotic food as a tool for empowerment across the value chain in Africa. Front. Microbiol..

[3] Doron S., Hibberd P.L., Goldin B., Thorpe C., McDermott L., Snydman D.R. (2015). Effect of *Lactobacillus rhamnosus* GG administration on vancomycin-resistant *Enterococcus* colonization in adults with comorbidities. Antimicrob. Agents Chemother..

[4] Yan F., Polk D.B. (2012). *Lactobacillus rhamnosus* GG: An Updated Strategy to Use Microbial Products to Promote Health. Funct. Food Rev..

[5] De Keersmaecker S.C.J., Verhoeven T.L.A., Desair J., Marchal K., Vanderleyden J., Nagy I. (2006). Strong antimicrobial activity of *Lactobacillus rhamnosus* GG against *Salmonella typhimurium* is due to accumulation of lactic acid. FEMS Microbiol. Lett..

[6] Princivalli M.S., Paoletti C., Magi G., Palmieri C., Ferrante L., Facinelli B. (2009). *Lactobacillus rhamnosus* GG inhibits invasion of cultured human respiratory cells by prtF1-positive macrolide-resistant group A streptococci. Lett. Appl. Microbiol..

[7] Ranadheera C., Vidanarachchi J., Rocha R., Cruz A., Ajlouni S. (2017). Probiotic Delivery through Fermentation: Dairy vs. Non-Dairy Beverages. Fermentation.

[8] Valero-Cases E., Cerdá-Bernad D., Pastor J.J., Frutos M.J. (2020). Non-dairy fermented beverages as potential carriers to ensure probiotics, prebiotics, and bioactive compounds arrival to the gut and their health benefits. Nutrients.

[9] Szparaga A., Tabor S., Kocira S., Czerwińska E., Kuboń M., Płóciennik B., Findura P. (2019). Survivability of probiotic bacteria in model systems of non-fermented and fermented coconut and hemp milks. Sustainability.

[10] Lipan L., Rusu B., Sendra E., Hernández F., Vázquez-Araújo L., Vodnar D.C., Carbonell-Barrachina Á.A. (2020). Spray drying and storage of probiotic-enriched almond milk: Probiotic survival and physicochemical properties. J. Sci. Food Agric..

[11] Su Y., Zheng X., Zhao Q., Fu N., Xiong H., Wu W.D., Chen X.D. (2019). Spray drying of *Lactobacillus rhamnosus* GG with calcium-containing protectant for enhanced viability. Powder Technol..

[12] Drozłowska E., Łopusiewicz Ł., Mężyńska M., Bartkowiak A. (2020). Valorization of flaxseed oil cake residual from cold-press oil production as a material for preparation of spray-dried functional powders for food applications as emulsion stabilizers. Biomolecules.

[13] Drozłowska E., Bartkowiak A., Trocer P., Kostek M., Tarnowiecka-Kuca A., Łopusiewicz Ł. (2021). Formulation and Evaluation of Spray-Dried Reconstituted Flaxseed Oil-In-Water Emulsions Based on Flaxseed Oil Cake Extract as Emulsifying and Stabilizing Agent. Foods.

[14] Azam M., Saeed M., Pasha I., Shahid M. (2020). A prebiotic-based biopolymeric encapsulation system for improved survival of *Lactobacillus rhamnosus*. Food Biosci..

[15] Lai K., How Y., Pui L. (2021). Microencapsulation of *Lactobacillus rhamnosus* GG with flaxseed mucilage using co-extrusion technique. J. Microencapsul..

[16] Comunian T.A., Drusch S., Brodkorb A. (2021). Advances of plant-based structured food delivery systems on the in vitro digestibility of bioactive compounds. Crit. Rev. Food Sci. Nutr..

[17] Liu J., Shim Y.Y., Tse T.J., Wang Y., Reaney M.J.T. (2018). Flaxseed gum a versatile natural hydrocolloid for food and non-food applications. Trends Food Sci. Technol..

[18] Samborska K., Boostani S., Geranpour M., Hosseini H., Dima C., Khoshnoudi-Nia S., Rostamabadi H., Falsafi S.R., Shaddel R., Akbari-Alavijeh S. (2021). Green biopolymers from by-products as wall materials for spray drying microencapsulation of phytochemicals. Trends Food Sci. Technol..

[19] Reque P.M., Brandelli A. (2021). Encapsulation of probiotics and nutraceuticals: Applications in functional food industry. Trends Food Sci. Technol..

[20] Azhar M.D., Hashib S.A., Ibrahim U.K., Rahman N.A. (2021). Development of carrier material for food applications in spray drying technology: An overview. Mater. Today Proc..

[21] Dimitrellou D., Kandylis P., Petrović T., Dimitrijević-Branković S., Lević S., Nedović V., Kourkoutas Y. (2016). Survival of spray dried microencapsulated *Lactobacillus casei* ATCC 393 in simulated gastrointestinal conditions and fermented milk. LWT Food Sci. Technol..

[22] Ying D., Sun J., Sanguansri L., Weerakkody R., Augustin M.A. (2012). Enhanced survival of spray-dried microencapsulated *Lactobacillus rhamnosus* GG in the presence of glucose. J. Food Eng..

[23] Avila-Reyes S.V., Garcia-Suarez F.J., Jiménez M.T., San Martín-Gonzalez M.F., Bello-Perez L.A. (2014). Protection of *L. rhamnosus* by spray-drying using two prebiotics colloids to enhance the viability. Carbohydr. Polym..

[24] Alvarado-Reveles O., Fernández-Michel S., Jiménez-Flores R., Cueto-Wong C., Vázquez-Moreno L., Montfort G.R.C. (2019). Survival and Goat Milk Acidifying Activity of *Lactobacillus rhamnosus* GG Encapsulated with Agave Fructans in a Buttermilk Protein Matrix. Probiotics Antimicrob. Proteins.

[25] Sunny-Roberts E.O., Knorr D. (2009). The protective effect of monosodium glutamate on survival of *Lactobacillus rhamnosus* GG and *Lactobacillus rhamnosus* E-97800 (E800) strains during spray-drying and storage in trehalose-containing powders. Int. Dairy J..

[26] Janiszewska-Turak E., Hornowska Ł., Pobiega K., Gniewosz M., Witrowa-Rajchert D. (2021). The influence of Lactobacillus bacteria type and kind of carrier on the properties of spray-dried microencapsules of fermented beetroot powders. Int. J. Food Sci. Technol..

[27] Golowczyc M.A., Silva J., Teixeira P., De Antoni G.L., Abraham A.G. (2011). Cellular injuries of spray-dried *Lactobacillus* spp. isolated from kefir and their impact on probiotic properties. Int. J. Food Microbiol..

[28] Jinapong N., Suphantharika M., Jamnong P. (2008). Production of instant soymilk powders by ultrafiltration, spray drying and fluidized bed agglomeration. J. Food Eng..

[29] Zouari A., Briard-Bion V., Schuck P., Gaucheron F., Delaplace G., Attia H., Ayadi M.A. (2020). Changes in physical and biochemical properties of spray dried camel and bovine milk powders. LWT.

[30] Aydar E.F., Tutuncu S., Ozcelik B. (2020). Plant-based milk substitutes: Bioactive compounds, conventional and novel processes, bioavailability studies, and health effects. J. Funct. Foods.

[31] Rasika D.M.D., Vidanarachchi J.K., Rocha R.S., Balthazar C.F., Cruz A.G., Sant’Ana A.S., Ranadheera C.S. (2021). Plant-based milk substitutes as emerging probiotic carriers. Curr. Opin. Food Sci..

[32] Pimentel T.C., da Costa W.K.A., Barão C.E., Rosset M., Magnani M. (2021). Vegan probiotic products: A modern tendency or the newest challenge in functional foods. Food Res. Int..

[33] Akbarbaglu Z., Mahdi Jafari S., Sarabandi K., Mohammadi M., Khakbaz Heshmati M., Pezeshki A. (2019). Influence of spray drying encapsulation on the retention of antioxidant properties and microstructure of flaxseed protein hydrolysates. Colloids Surf. B Biointerfaces.

[34] Ancuţa P., Sonia A. (2020). Oil press-cakes and meals valorization through circular economy approaches: A review. Appl. Sci..

[35] Drozłowska E., Bartkowiak A., Łopusiewicz Ł. (2020). Characterization of flaxseed oil bimodal emulsions prepared with flaxseed oil cake extract applied as a natural emulsifying agent. Polymers.

[36] Guo Q., Zhu X., Zhen W., Li Z., Kang J., Sun X., Wang S., Cui S.W. (2021). Rheological properties and stabilizing effects of high-temperature extracted flaxseed gum on oil/water emulsion systems. Food Hydrocoll..

[37] Drozłowska E., Bartkowiak A., Trocer P., Kostek M., Tarnowiecka-Kuca A., Bienkiewicz G., Łopusiewicz Ł. (2021). The influence of flaxseed oil cake extract on oxidative stability of microencapsulated flaxseed oil in spray-dried powders. Antioxidants.

[38] Łopusiewicz Ł., Drozłowska E., Siedlecka P., Mężyńska M., Bartkowiak A., Sienkiewicz M., Zielińska-Bliźniewska H., Kwiatkowski P. (2019). Development, Characterization, and Bioactivity of Non-Dairy Kefir-Like Fermented Beverage Based on Flaxseed Oil Cake. Foods.

[39] Łopusiewicz Ł., Drozłowska E., Tarnowiecka-Kuca A., Bartkowiak A., Mazurkiewicz-Zapałowicz K., Salachna P. (2020). Biotransformation of flaxseed oil cake into bioactive camembert-analogue using lactic acid bacteria, *Penicillium camemberti* and *Geotrichum candidum*. Microorganisms.

[40] Łopusiewicz Ł., Drozłowska E., Trocer P., Kostek M., Bartkowiak A., Kwiatkowski P. (2021). The development of novel probiotic fermented plant milk alternative from flaxseed oil cake using *Lactobacillus rhamnosus* GG acting as a preservative agent against pathogenic bacteria during short-term refrigerated storage. Emir. J. Food Agric..

[41] Khalloufi S., Corredig M., Goff H.D., Alexander M. (2009). Flaxseed gums and their adsorption on whey protein-stabilized oil-in-water emulsions. Food Hydrocoll..

[42] Bustamante M., Villarroel M., Rubilar M., Shene C. (2015). *Lactobacillus acidophilus* La-05 encapsulated by spray drying: Effect of mucilage and protein from flaxseed (*Linum usitatissimum* L.). LWT Food Sci. Technol..

[43] Verruck S., de Liz G.R., Dias C.O., de Mello Castanho Amboni R.D., Prudencio E.S. (2019). Effect of full-fat goat’s milk and prebiotics use on *Bifidobacterium* BB-12 survival and on the physical properties of spray-dried powders under storage conditions. Food Res. Int..

[44] Fang S., Qiu W., Mei J., Xie J. (2020). Effect of Sonication on the Properties of Flaxseed Gum Films Incorporated with Carvacrol. Int. J. Mol. Sci..

[45] Gutiérrez C., Rubilar M., Jara C., Verdugo M., Sineiro J., Shene C. (2010). Flaxseed and flaxseed cake as a source of compounds for food industry. J. Soil Sci. Plant Nutr..

[46] Oomah B.D., Mazza G. (2001). Optimization of a spray drying process for flaxseed gum. Int. J. Food Sci. Technol..

[47] Razavi S., Janfaza S., Tasnim N., Gibson D.L., Hoorfar M. (2021). Microencapsulating polymers for probiotics delivery systems: Preparation, characterization, and applications. Food Hydrocoll..

[48] Bustamante M., Laurie-Martínez L., Vergara D., Campos-Vega R., Rubilar M., Shene C. (2020). Effect of three polysaccharides (inulin, and mucilage from chia and flax seeds) on the survival of probiotic bacteria encapsulated by spray drying. Appl. Sci..

[49] Bustamante M., Oomah B.D., Rubilar M., Shene C. (2017). Effective *Lactobacillus plantarum* and *Bifidobacterium infantis* encapsulation with chia seed (*Salvia hispanica* L.) and flaxseed (*Linum usitatissimum* L.) mucilage and soluble protein by spray drying. Food Chem..

[50] Horwitz W. (2000). Official Methods of Analysis of AOAC International.

[51] European Pharmacopoeia (2011). Flow Powder.

[52] Gong K.J., Shi A.M., Liu H.Z., Liu L., Hu H., Adhikari B., Wang Q. (2015). Emulsifying properties and structure changes of spray and freeze-dried peanut protein isolate. J. Food Eng..

[53] Joly C., Gay-Quéheillard J., Léké A., Chardon K., Delanaud S., Bach V., Khorsi-Cauet H. (2013). Impact of chronic exposure to low doses of chlorpyrifos on the intestinal microbiota in the Simulator of the Human Intestinal Microbial Ecosystem (SHIME^®^) and in the rat. Environ. Sci. Pollut. Res..

[54] Sumeri I., Arike L., Adamberg K., Paalme T. (2008). Single bioreactor gastrointestinal tract simulator for study of survival of probiotic bacteria. Appl. Microbiol. Biotechnol..

[55] Marzorati M., Vanhoecke B., De Ryck T., Sadaghian Sadabad M., Pinheiro I., Possemiers S., Van Den Abbeele P., Derycke L., Bracke M., Pieters J. (2014). The HMI^TM^ module: A new tool to study the Host-Microbiota Interaction in the human gastrointestinal tract in vitro. BMC Microbiol..

[56] Oomen A.G., Rompelberg C.J.M., Bruil M.A., Dobbe C.J.G., Pereboom D.P.K.H., Sips A.J.A.M. (2003). Development of an *in vitro* digestion model for estimating the bioaccessibility of soil contaminants. Arch. Environ. Contam. Toxicol..

[57] Bondue P., Lebrun S., Taminiau B., Everaert N., LaPointe G., Crevecoeur S., Daube G., Delcenserie V. (2020). A toddler SHIME^®^ model to study microbiota of young children. FEMS Microbiol. Lett..

[58] Van Den Abbeele P., Marzorati M., Derde M., De Weirdt R., Joan V., Possemiers S., Van De Wiele T. (2016). Arabinoxylans, inulin and *Lactobacillus reuteri* 1063 repress the adherent-invasive *Escherichia coli* from mucus in a mucosa-comprising gut model. NPJ Biofilms Microbiomes.

[59] Van den Abbeele P., Roos S., Eeckhaut V., MacKenzie D.A., Derde M., Verstraete W., Marzorati M., Possemiers S., Vanhoecke B., Van Immerseel F. (2012). Incorporating a mucosal environment in a dynamic gut model results in a more representative colonization by lactobacilli. Microb. Biotechnol..

[60] Rahman M.M., Kim W.S., Kumura H., Shimazaki K.I. (2008). Autoaggregation and surface hydrophobicity of bifidobacteria. World J. Microbiol. Biotechnol..

[61] Ranadheera C.S., Evans C.A., Adams M.C., Baines S.K. (2015). Microencapsulation of *Lactobacillus acidophilus* LA-5, *Bifidobacterium animalis* subsp. lactis BB-12 and *Propionibacterium jensenii* 702 by spray drying in goat’s milk. Small Rumin. Res..

[62] Ananta E., Volkert M., Knorr D. (2005). Cellular injuries and storage stability of spray-dried *Lactobacillus rhamnosus* GG. Int. Dairy J..

[63] Deshwal G.K., Singh A.K., Kumar D., Sharma H. (2020). Effect of spray and freeze drying on physico-chemical, functional, moisture sorption and morphological characteristics of camel milk powder. LWT.

[64] Rajam R., Anandharamakrishnan C. (2015). Microencapsulation of *Lactobacillus plantarum* (MTCC 5422) with fructooligosaccharide as wall material by spray drying. LWT Food Sci. Technol..

[65] Moayyedi M., Eskandari M.H., Rad A.H.E., Ziaee E., Khodaparast M.H.H., Golmakani M.T. (2018). Effect of drying methods (electrospraying, freeze drying and spray drying) on survival and viability of microencapsulated *Lactobacillus rhamnosus* ATCC 7469. J. Funct. Foods.

[66] Wilson W.W., Wade M.M., Holman S.C., Champlin F.R. (2001). Status of methods for assessing bacterial cell surface charge properties based on zeta potential measurements. J. Microbiol. Methods.

[67] Telang A.M., Thorat B.N. (2010). Optimization of process parameters for spray drying of fermented soy milk. Dry. Technol..

[68] Maciel G.M., Chaves K.S., Grosso C.R.F., Gigante M.L. (2014). Microencapsulation of *Lactobacillus acidophilus* La-5 by spray-drying using sweet whey and skim milk as encapsulating materials. J. Dairy Sci..

[69] Dimitrellou D., Salamoura C., Kontogianni A., Katsipi D., Kandylis P., Zakynthinos G., Varzakas T. (2019). Effect of milk type on the microbiological, physicochemical and sensory characteristics of probiotic fermented milk. Microorganisms.

[70] Safdar B., Pang Z., Liu X., Jatoi M.A., Mehmood A., Rashid M.T., Ali N., Naveed M. (2019). Flaxseed gum: Extraction, bioactive composition, structural characterization, and its potential antioxidant activity. J. Food Biochem..

[71] Chung M.W.Y., Lei B., Li-Chan E.C.Y. (2005). Isolation and structural characterization of the major protein fraction from NorMan flaxseed (*Linum usitatissimum* L.). Food Chem..

[72] Chen C., Chi Y.J., Xu W. (2012). Comparisons on the Functional Properties and Antioxidant Activity of Spray-Dried and Freeze-Dried Egg White Protein Hydrolysate. Food Bioprocess Technol..

[73] Ritter P., Kohler C., Von Ah U. (2009). Evaluation of the passage of *Lactobacillus gasseri* K7 and bifidobacteria from the stomach to intestines using a single reactor model. BMC Microbiol..

[74] Basiri S., Haidary N., Shekarforoush S.S., Niakousari M. (2018). Flaxseed mucilage: A natural stabilizer in stirred yogurt. Carbohydr. Polym..

[75] Laparra J.M., Sanz Y. (2009). Comparison of in vitro models to study bacterial adhesion to the intestinal epithelium. Lett. Appl. Microbiol..

[76] Yadav R., Vij R., Kapila S., Khan S.H., Kumar N., Meena S., Kapila R. (2019). Milk fermented with probiotic strains *Lactobacillus rhamnosus* MTCC: 5957 and *Lactobacillus rhamnosus* MTCC: 5897 ameliorates the diet-induced hypercholesterolemia in rats. Ann. Microbiol..

[77] Sivieri K., Morales M.L.V., Adorno M.A.T., Sakamoto I.K., Saad S.M.I., Rossi E.A. (2013). Lactobacillus acidophilus CRL 1014 improved “gut health” in the SHIME^®^ reactor. BMC Gastroenterol..

[78] Tsai C.C., Lin P.P., Hsieh Y.M., Zhang Z.Y., Wu H.C., Huang C.C. (2014). Cholesterol-Lowering Potentials of Lactic Acid Bacteria Based on Bile-Salt Hydrolase Activity and Effect of Potent Strains on Cholesterol Metabolism In Vitro and In Vivo. Sci. World J..

